# Effects of acupuncture at the Taichong (LIV3) and Hegu (LI4) points on functional connectivity with the retrosplenial cortex in patients with Alzheimer’s disease

**DOI:** 10.3389/fnins.2024.1511183

**Published:** 2025-01-14

**Authors:** Junkai Wang, Xinyue Bai, Xiaojun Chen, Shui Liu, Mengmeng Sun, Kuncheng Li, Yunsong Zheng, Zhiqun Wang

**Affiliations:** ^1^Department of Radiology, Aerospace Center Hospital, Beijing, China; ^2^School of Psychology, Capital Normal University, Beijing, China; ^3^Department of Traditional Chinese Medicine, Aerospace Center Hospital, Beijing, China; ^4^Department of Radiology, Xuanwu Hospital of Capital Medical University, Beijing, China; ^5^Key Laboratory of Acupuncture and Medicine of Shaanxi Province, Shaanxi University of Chinese Medicine, Xianyang, China

**Keywords:** Alzheimer’s disease, acupuncture, resting-state functional magnetic resonance imaging, retrosplenial cortex, regulatory effects

## Abstract

**Background:**

Acupuncture has been demonstrated to have a promising effect on Alzheimer’s disease (AD), but the underlying neural mechanisms remain unclear. The retrosplenial cortex (RSC) is one of the earliest brain regions affected in AD, and changes in its functional connectivity (FC) are reported to underlie disease-associated memory impairment. The aim of this study was to examine the effect of acupuncture on FC with the RSC in patients with AD.

**Methods:**

Demographic data, neuropsychological assessments, and resting-state functional magnetic resonance imaging (fMRI) data were collected from 14 AD patients and 14 normal controls (NCs) matched by age, sex, and educational level at baseline. After the baseline MRI scan, acupuncture stimulation on the Taichong (LIV3) and Hegu (LI4) points was performed for 3 min. Then, another 10 min of fMRI data were acquired after the needle was withdrawn. A dataset that included 100 healthy participants was also included to construct a reliable FC map of the RSC. Two sets of regions of interest (ROIs) in the RSC were selected to assess the sustained effect of acupuncture on FC with the RSC in AD patients and NCs.

**Results:**

Two sets of RSC ROI-based analyses demonstrated robust positive connectivity with the hippocampus (HPC). Furthermore, multiple brain regions, including the bilateral thalamus, bilateral posterior cingulate cortex (PCC), bilateral subcallosal cingulate gyrus (SCG), bilateral orbitofrontal cortex (OFC), and right precuneus, showed decreased FC with the RSC in the AD group and increased FC with the RSC in the NC group after acupuncture compared to that at baseline. Acupuncture also specifically elicited increased FC between the RSC and the HPC as well as between the RSC and the parahippocampal gyrus in AD patients and decreased FC between the RSC and the visual cortices in NCs. Additionally, diminished FC with the RSC was correlated with neuropsychological scale scores in the AD group before acupuncture treatment.

**Conclusion:**

These findings confirm and extend previous studies suggesting that acupuncture at Taichong (LIV3) and Hegu (LI4) can exert bidirectional and benign regulatory effects on RSC connectivity in AD patients.

## Introduction

Alzheimer’s disease (AD) is a progressive neurodegenerative disorder and represents the most common type of dementia, accounting for 60–80% of all dementia cases (Alzheimer’s Association Report, 2022). AD is characterized by initial memory impairment and progressive cognitive decline, as well as decline in language, attention, and executive function. Currently, more than 50 million people worldwide live with dementia, with this number expected to reach approximately 139 million by 2050 (Livingston et al., 2020; World Health Organization, 2021). However, there are still no effective preventive or therapeutic approaches for AD, and early diagnosis and prevention of AD in its prodromal stage are priorities.

A battery of treatment strategies for AD has been explored (Rajendran and Krishnan, 2024; Chen et al., 2024), among which acupuncture has been widely applied to treat neurodegenerative disease considering its effectiveness and limited side effects (Chen et al., 2004). As an important complementary and alternative treatment to traditional Chinese medicine, acupuncture has been demonstrated to have a promising effect on AD through multiple mechanisms, such as decreasing amyloid-β protein levels, reducing neuroinflammation, enhancing the antioxidant defense system, and regulating brain energy metabolism (Teja et al., 2021; Wu et al., 2023; Yin et al., 2021). Especially, animal studies have proved that acupuncture reduced Aβ deposits in neurons through an augmented autophagy pathway (Teja et al., 2021). Acupuncture can also reduce the expression of interleukin-1beta (IL-1β) and IL-6 and regulate reactive oxygen species (ROS) related signaling pathways, suggesting therapeutic role by inhibiting the specific inflammatory reaction and ameliorating neuronal antioxidant damage (Wu et al., 2023). According to traditional Chinese medicine theories, points are sites of acupuncture manipulation and the basis for the mechanism of acupuncture. Therefore, point selection may impact acupuncture efficacy in AD treatment. Taichong (LIV3) and Hegu (LI4) are located at source points, making them important in clinical therapy. Source points (also called Yuan points) refer to the places where the Yuan Qi resides, surfaces, and lingers, which are hubs for internal and external energies gathering and transforming. Clinically, these points are of great significance in treating diseases of the internal organs. LIV3 and LI4 are collectively aware of the famous acupoint combination named the Si Guan (The Four Gates) or Four Bars, which are thought to promote the circulation of Qi and blood throughout the body and have a calming effect. Acupuncture at the Si Guan points has been proven to be effective in ameliorating AD symptoms (Yin et al., 2021). However, the potential neural mechanisms underlying their effectiveness remain limited. Previous studies have used advanced neuroimaging methods, such as resting-state functional magnetic resonance imaging (rs-fMRI), to assess the effect of acupuncture at the Si Guan points for the treatment of AD and explore the potential mechanisms of acupuncture therapy for AD (Wang et al., 2014; Liang et al., 2014; Zheng et al., 2018; Ji et al., 2021). These studies revealed that acupuncture at the Si Guan points can modulate functional activity and connectivity within cortical-subcortical functional networks, such as the default mode network (DMN), hippocampal functional network, frontal parietal network, and sensorimotor network (Wang et al., 2014; Liang et al., 2014; Zheng et al., 2018; Ji et al., 2021). Specifically, compared to the pre-acupuncture condition, increased activity and connectivity within hippocampal functional network and decreased activity and connectivity in brain regions of frontal parietal network and sensorimotor network were identified in AD patients after acupuncture (Wang et al., 2014; Liang et al., 2014; Zheng et al., 2018; Ji et al., 2021), indicating the benefits of brain plasticity after acupuncture treatment.

Within the DMN subregions, the retrosplenial cortex (RSC) is one of the earliest brain regions affected in AD, as it appears to be vulnerable to amyloid pathology in prodromal AD, and its dysfunction is reported to underlie disease-associated memory impairment (Buckner et al., 2005; Trask and Fournier, 2022). The RSC is considered a part of the posterior cingulate cortex (PCC) (Vann et al., 2009). However, the PCC is anatomically and functionally heterogeneous, and the RSC is typically functionally misattributed to other parts of the PCC due to their adjacent anatomical locations (Vann et al., 2009; Epstein et al., 2007). Recent studies have indicated the importance of distinguishing the RSC from the PCC and also demonstrated that the RSC forms a critical gateway between the hippocampus (HPC) and other brain regions to maintain communication between each other and support episodic memory (Dillen et al., 2016; Kaboodvand et al., 2018). More recently, it has been reported that the functional connectivity (FC) between the RSC and the HPC is linked to neocortical tau accumulation and that these measures may play critical roles in AD-related memory loss (Ziontz et al., 2021). Given the crucial role of the RSC in the progression of AD-related memory and cognitive impairment, it is of great interest to investigate the modulatory effect of acupuncture at the Si Guan points on FC with the RSC in individuals with AD. To the best of our knowledge, little is known about this research question.

Therefore, the aim of this study was to explore the modulatory effect of acupuncture at the Si Guan points on RSC FC in AD patients compared to that in healthy controls. Reliable intrinsic FC with the RSC was first explored based on a dataset that included 100 healthy participants. Then, the interaction effect of acupuncture by group masked by the above-generated RSC connectivity map was examined to identify brain regions in AD patients and healthy controls that could be modulated by acupuncture. It is hypothesized that acupuncture at the Si Guan points could produce bidirectional modulatory effects on FC between the RSC and brain regions related to the DMN, hippocampal functional network, and frontal parietal network in AD patients.

## Materials and methods

### Participants and data acquisition—healthy participants

The current study included 100 healthy participants. All participants were recruited from the local community by using advertisements. Written informed consent was obtained from all of the participants after the study had been fully explained. The study was approved by the Medical Research Ethics Committee of Aerospace Center Hospital. Recruitment took place from September 1, 2022, and completed on July 30, 2023. The inclusion criteria for healthy participants were cognitive health without memory complaints and a Mini-Mental State Examination (MMSE) (Folstein et al., 1975) score of 28 or higher. The exclusion criteria for all participants were as follows: history of head trauma or cranial surgery, history of neurological or psychiatric disorders, or any contraindications to magnetic resonance image (MRI).

Magnetic resonance images were acquired on a MAGNETOM Prisma 3 T MR scanner (Siemens Medical Solutions, Erlangen, Germany) with a 64-channel head-neck coil. High-resolution T1-weighted anatomical images were acquired for functional image registration and localization by using a 3D magnetization-prepared rapid gradient echo (MPRAGE) sequence with the following parameters: repetition time (TR) = 2,530 ms, echo time (TE) = 2.98 ms, inversion time (TI) = 1,100 ms, flip angle (FA) = 7°, field of view (FOV) = 224 mm × 256 mm, acquisition matrix = 224 × 256, interpolated to 448 × 512, 192 continuous sagittal slices, slice thickness (ST) = 1 mm, and voxel size = 0.5 × 0.5 × 1 mm^3^. High-resolution resting-state functional images of the whole brain were axially acquired using a simultaneous multislice echoplanar imaging sequence. The scanning plane was parallel to the straight gyrus, and the phase encoding direction was from posterior to anterior, with the following parameters: TR = 2,000 ms, TE = 30 ms, FA = 90°, matrix size = 112 × 112, FOV = 224 mm × 224 mm, ST = 2 mm, gap = 0.3 mm, 62 slices, voxel size = 2 × 2 × 2 mm^3^, and multiband factor = 2. Field map imaging was performed with a double-echo gradient echo sequence with the following parameters: TR = 620 ms, TE = 4.92/7.38 ms, voxel size = 2 × 2 × 2 mm^3^, FA = 60°, matrix size = 112 × 112, gap = 0.3 mm, and 62 slices. The field map image was computed from the two-phase images and one magnitude image. Suitable foam padding and earplugs were used to limit head motion and minimize the noise of the scanner. During the MRI session, participants were instructed to hold still, keep their eyes closed, and refrain from thinking of anything in particular.

### Participants and data acquisition—acupuncture group

This dataset is from a previously published study (Wang et al., 2014). Twenty-eight right-handed subjects participated in the current study, including 14 patients with AD and 14 age-, sex-, and education-matched normal controls (NCs). The AD participants included individuals who had consulted a memory clinic at Xuanwu Hospital with memory complaints. The NCs were recruited from the local community. The experimental procedure was approved by the Medical Research Ethics Committee of Xuanwu Hospital, Capital Medical University. Written informed consent was obtained from all participants or their legal guardians after the study had been fully explained.

All AD patients were screened by research clinicians according to the inclusion and exclusion criteria. The diagnosis of AD was based on the Diagnostic and Statistical Manual of Mental Disorders-Fifth Edition criteria for Alzheimer’s Dementia and the National Institutes on Aging and the Alzheimer’s Association on diagnostic guidelines for AD (McKhann et al., 2011). The NCs had no history of neurological disease or cognitive dysfunction. The exclusion criteria for all of the participants included the following: (1) cognitive impairment caused by head trauma or cranial surgery; (2) a history of neurological or psychiatric disorders that could cause cognitive impairments; and (3) contraindications to MRI.

All of the participants underwent cognitive assessments, including the Clinical Dementia Rating (CDR) (Morris, 1993), the MMSE, and the Montreal Cognitive Assessment (MoCA) (Nasreddine et al., 2005), as screening measures. Memory function was also measured using the Auditory Verbal Learning Test (AVLT) (Zhao et al., 2012). All demographic and neuropsychological assessment results are shown in Table 1.

**Table 1:** Demographic and neuropsychological assessments of participants.

	AD (*n* = 14)	NC (*n* = 14)	*p*-value
Age, y	66.93 (8.91)	66.07 (5.78)	0.77^a^
Sex, males/females	4/10	6/8	0.16^b^
Education, y	10.07 (3.38)	11.00 (4.52)	0.54^a^
MMSE scores	15.93 (4.12)	28.00 (1.41)	**<0.001** ^a^
MoCA scores	13.07 (4.05)	26.64 (2.02)	**<0.001** ^a^
AVLT scores	17.36 (6.28)	50.64 (9.01)	**<0.001** ^a^
Immediate recall	11.36 (3.95)	26.86 (5.25)	**<0.001** ^a^
Delayed recall	2.64 (1.60)	11.07 (2.76)	**<0.001** ^a^
Recognition	3.36 (1.55)	12.71 (2.09)	**<0.001** ^a^
CDR, (0, 0.5, 1–2)	0.5 = 1, 1–2 = 13	0 = 14	**<0.001** ^b^
Framewise displacement^c^	0.16 (0.067)	0.16 (0.074)	0.87^a^

Results are given as means (standard deviation, SD). Independent sample *t*-test and chi-square analyses were applied to test for group differences, statistical significance level was set at *p* < 0.05 (two-tailed). Significant *p*-values were in bold. MMSE, Mini-Mental State Examination; MoCA, Montreal Cognitive Assessment; AVLT, Auditory Verbal Learning Test; CDR, Clinical Dementia Rating; AD, Alzheimer’s disease; NC, normal controls.

a The *p*-value was obtained using independent sample t-test.

b The *p*-value was obtained using Chi-square test.

c One subject was not included due to poor image quality (AD = 1).

The MR data were acquired using a Siemens Verio 3-Tesla scanner (Siemens, Erlangen, Germany). High-resolution T1-weighted anatomical images were acquired using a 3D MPRAGE sequence. The acquisition parameters were as follows: TR = 1,900 ms, TE = 2.22 ms, TI = 900 ms, FA = 9°, acquisition matrix = 256 × 256, 176 continuous sagittal slices, ST = 1 mm, and voxel size = 1 × 1 × 1 mm^3^. Resting-state functional imaging was obtained in an axial orientation using a gradient-echo echo-planar imaging (EPI) sequence with the following parameters: TR = 3,000 ms, TE = 30 ms, FA = 90°, matrix size = 64 × 64, FOV = 224 mm × 224 mm, ST = 3 mm, gap = 0.48 mm, 43 slices.

### Experimental design

Details on the experimental design can be found in the study of Wang et al. (2014). Briefly, the baseline resting-state fMRI data were first acquired during the initial 3 min. Then, all participants received manual acupuncture at bilateral Taichong (LIV3, located on the dorsum of the left and right feet) and Hegu (LI4, located on the dorsum of the left and right hands) points for the following 3 min. After the needles were withdrawn, another 10 min of resting-state fMRI data were acquired.

### Image preprocessing

All image data were preprocessed using Statistical Parametric Mapping (SPM 12, University College London, London, United Kingdom; http://www.fil.ion.ucl.ac.uk/spm/software/spm12) and the CONN-fMRI functional connectivity toolbox v22a [http://www.nitrc.org/projects/conn (Whitfield-Gabrieli and Nieto-Castanon, 2012)].

Briefly, the first 10 volumes of each subject were discarded to allow for signal stabilization. The remaining volumes were slice-time corrected, and images with head motion exceeding 2.5 mm in any direction or 2.5° rotational movement were excluded from further analysis (one case of AD was excluded for this reason). The average framewise displacement (FD) was calculated and tested for significant differences between groups. Moreover, for the resting-state fMRI data of 100 healthy participants, field map correction was applied to correct for distortion caused by field inhomogeneities within the scanner. The magnitude images and the phase images were used for susceptibility distortion correction (SDC) as part of the unwarp step, where the functional data were resampled along the phase encoding direction of EPI acquisition (posterior-anterior). To spatially normalize the fMRI data, the mean functional images were coregistered to structural images. The resulting aligned structural images were normalized to standard Montreal neurological institute (MNI) space and segmented into gray matter (GM), white matter (WM), and cerebrospinal fluid (CSF) tissue classes using the SPM12 unified segmentation and normalization procedure. The same estimated nonlinear transformation that was obtained from this step was subsequently applied to the functional data. Then, the functional data were smoothed with a Gaussian kernel of 6 × 6 × 6 mm^3^ full width at half maximum (FWHM) to reduce spatial noise. Using the CONN toolbox, potential confounding effects, such as noise components from cerebral WM and cerebrospinal areas were estimated and regressed out using the anatomical component base noise reduction strategy (aCompCor) (Behzadi et al., 2007). The estimated subject-motion parameters, and identified outlier scans (Power et al., 2014), were also regressed out. Finally, the BOLD time series were simultaneously filtered with the recommended bandpass filter (below 0.008 Hz or above 0.09 Hz) (Weissenbacher et al., 2009) followed by linear detrending to minimize the effect of low-frequency drifts and the influence of physiology, head motion and other noise sources (Soares et al., 2016)

### Definition of regions of interest

**Figure 1:**
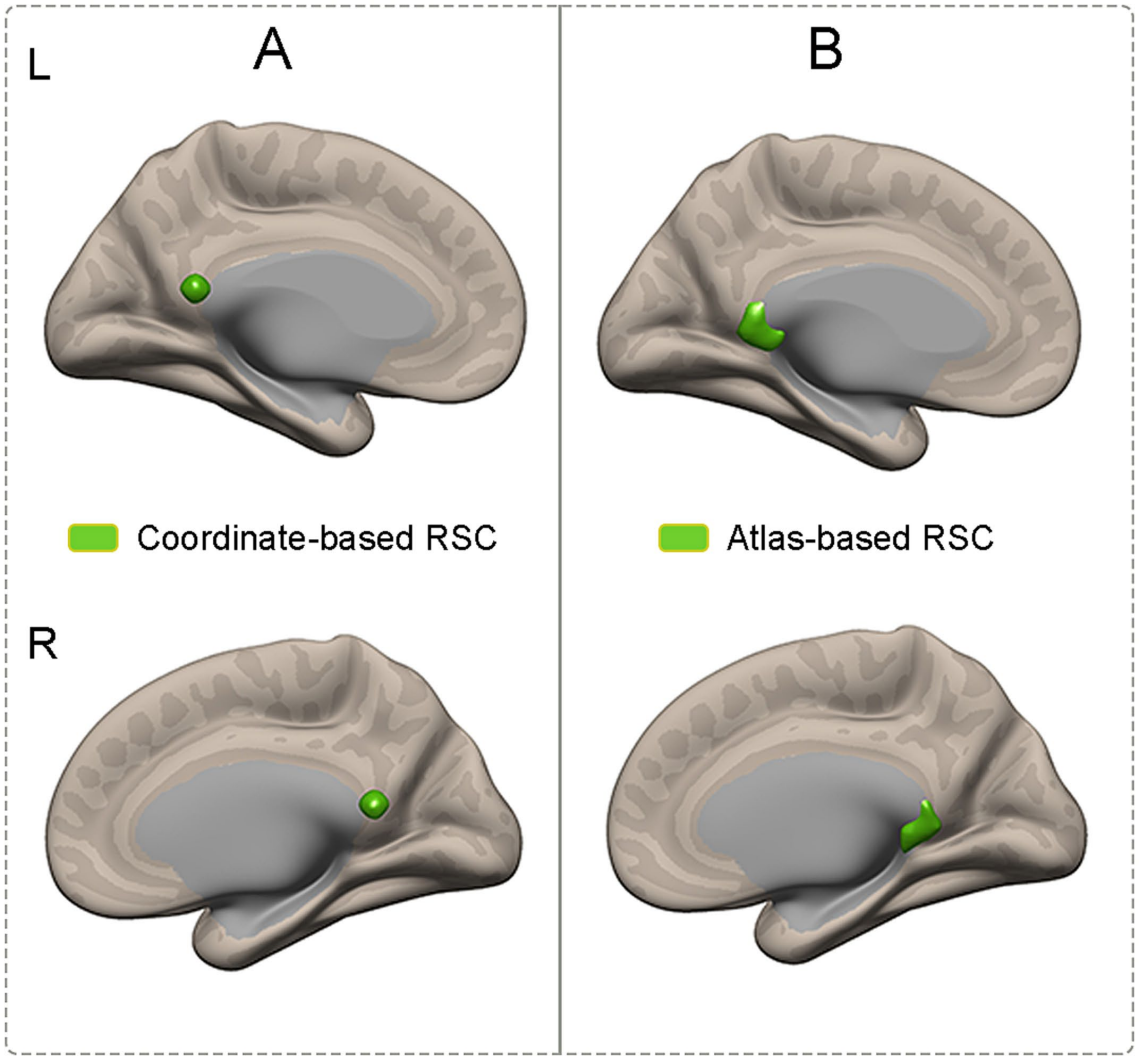
Illustration of ROIs which were obtained based on coordinates and atlas. **(A)** The RSC was defined according to the coordinates. **(B)** The RSC was defined according to the atlas. RSC, retrosplenial cortex; ROIs, region of interests.

In this study, to ensure the repeatability and reliability of the results of connectivity with the RSC, two sets of region-of-interest (ROI)-based analyses were performed. One set of ROIs was obtained based on a probabilistic atlas. The bilateral RSC was defined according to the Brainnetome Atlas (Brainnetome Atlas Viewer, vision 1.0.1, http://atlas.brainnetome.org/) (Fan et al., 2016). Specifically, the bilateral RSC was modified in location according to the Brainnetome atlas to verify the corresponding location with a probability of 50% of being located within the RSC (Figure 1B). The other set of ROIs was defined according to the MNI coordinates. A spherical seed (7 mm in radius) in the RSC (MNI coordinates: 2, −52, 16) (Figure 1A) was defined based on previously published literature (Dillen et al., 2016).

### Functional connectivity analysis

After data preprocessing, two sets of RSC ROI-based analyses were performed to identify FC with the RSC. The mean time series of the RSC ROIs were extracted from the processed functional data of each participant. Then, RSC-based connectivity maps were generated for each participant by calculating Pearson’s correlation coefficients between the mean signal of the RSC ROI and the time courses of each voxel in the whole brain. Each correlation map was converted into a *z* value map by using Fisher’s *r*-to-*z* transformation. Afterward, to generate a reliable FC map of the RSC, one-sample *t*-tests were separately performed for 100 healthy participants on the correlation maps of the RSC ROIs. The significance level was set at a cluster level of *p* < 0.05, which was corrected for multiple comparisons by using familywise error (FWE). The significant results of the RSC FC were then saved as the masks for subsequent group-level analysis.

To determine the effect of acupuncture on RSC FC in the AD patients and NC group, a voxelwise 2 × 2 two-way repeated measure analysis of variance (ANOVA) was conducted on participants’ connectivity maps of the RSC by using the abovementioned created masks for Pre_AD (AD group before acupuncture treatment), Pre_NC (NC group before acupuncture treatment), Post_AD (AD group after acupuncture treatment), and Post_NC (NC group after acupuncture treatment). In this study, we were only interested in the interaction effect of acupuncture treatment by group, which represents the true effect of acupuncture stimulation. If the interaction effect was significant, the *post hoc* independent two-sample *t*-test was used to compare all simple effects, as the *F* test is nondirectional.

### Statistical analyses

The independent sample *t*-test and two-tailed chi-square test were used to compare demographic features and neuropsychological scores between the AD patients and NCs by using the IBM SPSS Statistics for Windows, version 22.0 (IBM Corp., Armonk, NY, United States).

The interaction effect of acupuncture treatment by group was determined using two-way repeated ANOVA within the aforementioned masks of reliable FC connectivity with the RSC after regressing out the influence of age, education years, FD values, and total intracranial volume (TIV). The interaction effect map of acupuncture treatment by group was first generated, and Gaussian random field (GRF) theory was used for cluster-level multiple comparison correction (voxel *p*-value <0.005; cluster *p*-value <0.05). *Post hoc* comparisons were used to identify differences in RSC functional connectivity between the AD and HC groups before and after acupuncture treatment.

To investigate the relationship between the FC alterations and cognitive ability, the averaged FC strengths (indicated by *z* values) were extracted. Specifically, after significant interaction effect results of acupuncture treatment by group were determined, the brain areas which showed significant interaction effect was saved as masks. Then, the averaged *z* values of the FC between the RSC and the abovementioned significant brain areas were extracted for the following analysis. After that, Pearson’s correlation analysis was conducted to explore the relationship between the behavioral data and the RSC FC with a significant interaction effect in patients with AD before acupuncture treatment. A *p*-value <0.05 was considered to indicate statistical significance.

## Results

### Demographic data and neuropsychological assessments

The demographic data and neuropsychological assessments of the 24 participants who underwent MRI are listed in Table 1. Participants in the AD and NC groups were well matched for age, sex, and years of education. As expected, there were significant differences in cognitive performance between the two groups. Compared with the NCs, the individuals in the AD group had lower scores on the MMSE, MoCA, CDR, and AVLT measures, including immediate recall (AVLT_IR), delayed recall (AVLT_DR), and recognition (all *p* < 0.001). After inspection for excessive motion artifacts, one AD patient was excluded from the study due to head motion. No significant difference was observed in FD values between the AD and NC groups, (*p* = 0.87).

### Identification of RSC functional connectivity

**Figure 2:**
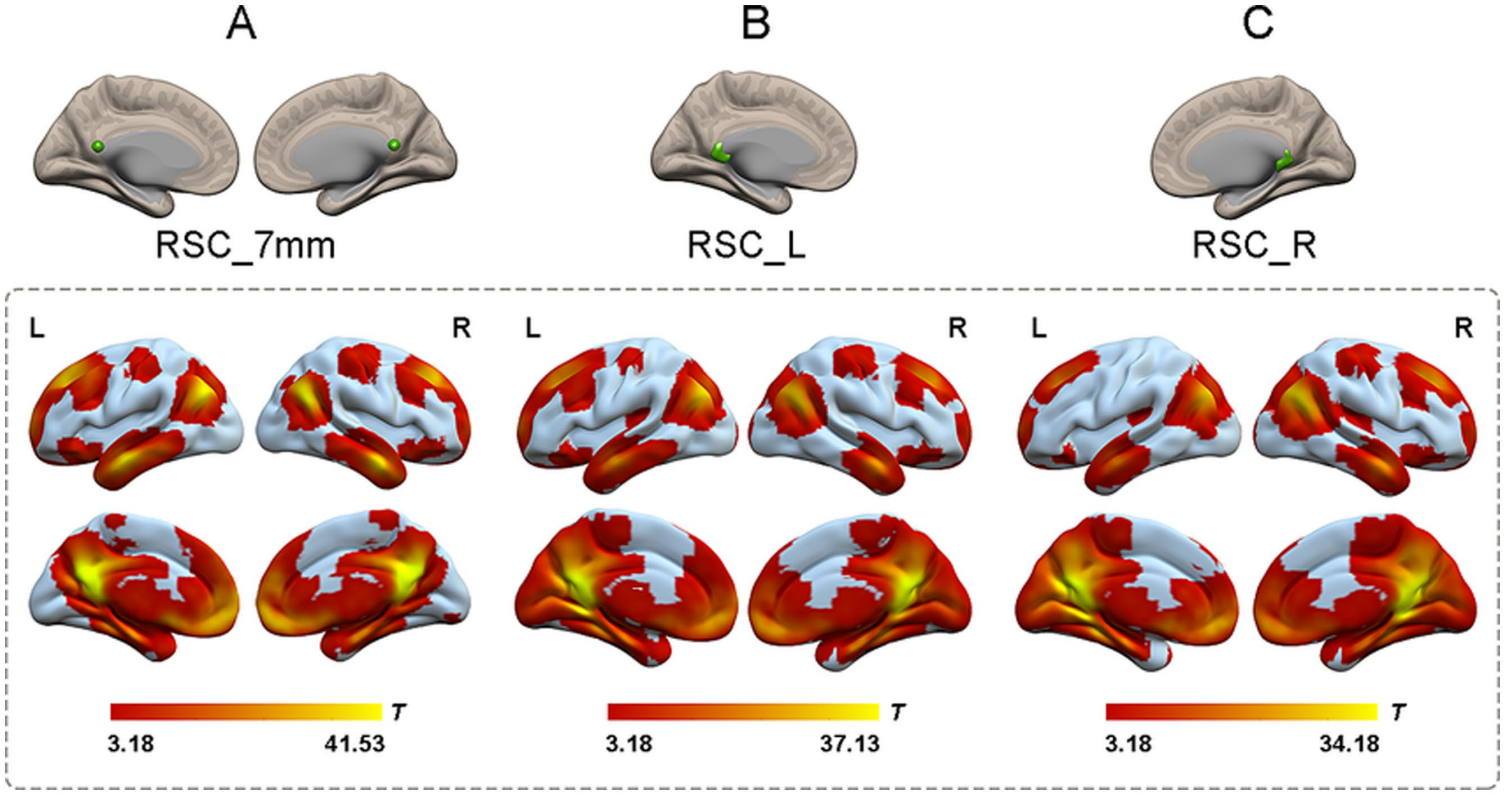
The one-sample *t*-test of functional connectivity patterns of RSC in 100 healthy participants. **(A)** The functional connectivity patterns of RSC based on coordinates. The RSC (green color) showed significantly positive functional connectivity with the PCC, ventral and dorsal medial prefrontal cortex, lateral temporal cortex, and hippocampal formation (shown in warm colors; *p* < 0.05, cluster-level FWE corrected). **(B)** The functional connectivity patterns of left RSC based on atlas. Left RSC showed robust positive connectivity with multiple brain areas including the PCC, ventral and dorsal medial prefrontal cortex, lateral temporal cortex, and hippocampal formation (shown in warm colors; *p* < 0.05, cluster-level FWE corrected). **(C)** The functional connectivity patterns of right RSC based on atlas. Right RSC and left RSC showed highly similar functional connectivity patterns (shown in warm colors; *p* < 0.05, cluster-level FWE corrected). RSC, retrosplenial cortex; L, left; R, right.

To generate reliable RSC connectivity, the spatial distribution of the RSC connectivity maps was obtained by using correlational maps of two sets of ROIs from the dataset that contained 100 healthy participants. Both sets of RSC ROIs showed robust positive connectivity with multiple brain areas, including the PCC, ventral and dorsal medial prefrontal cortex, lateral temporal cortex, and hippocampal formation, which replicated a classical connectivity pattern of the DMN (Figure 2).

### The effect of acupuncture on RSC connectivity in the AD and NC groups—coordinate-based ROIs

**Figure 3:**
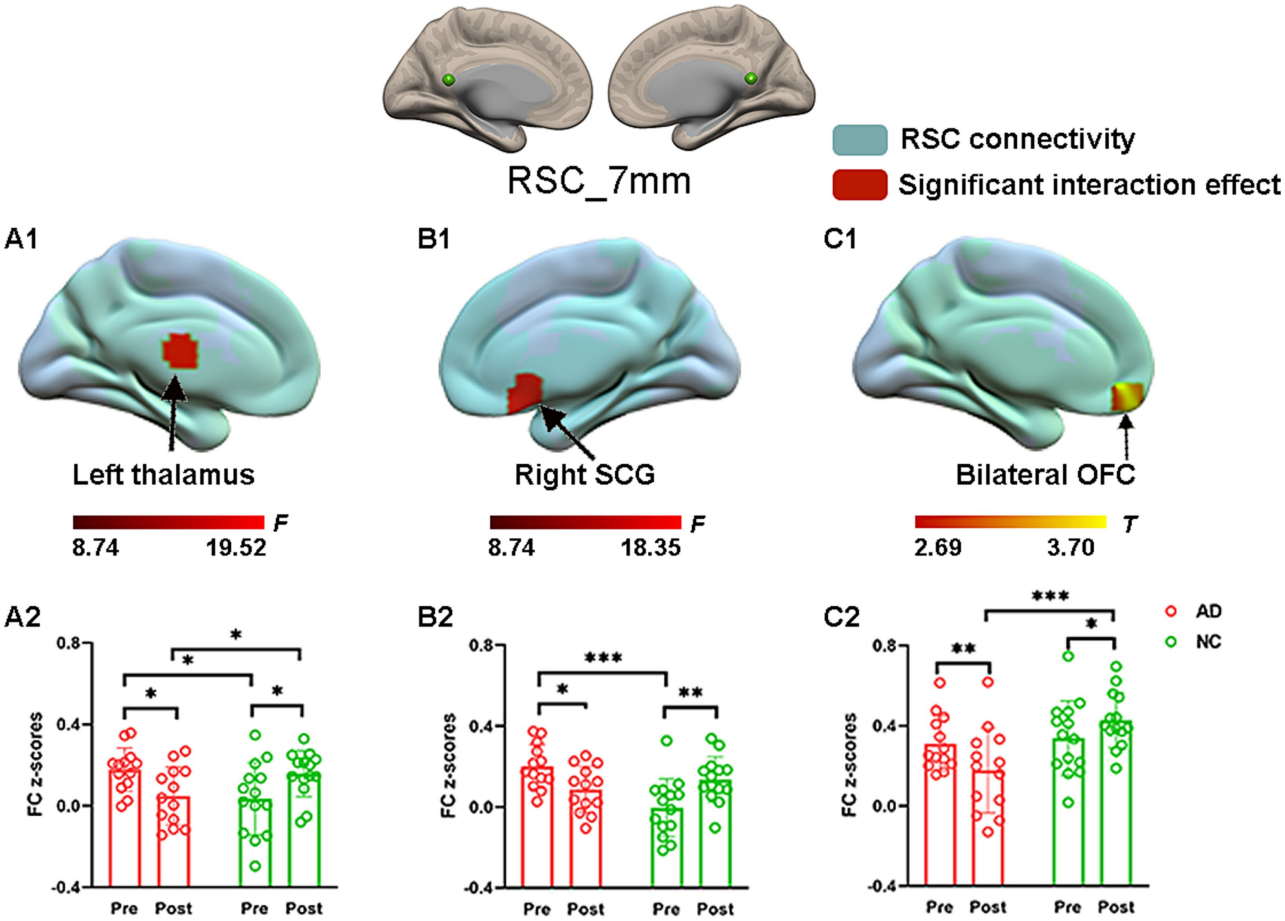
The effect of acupuncture on RSC connectivity in the AD and NC groups as revealed by coordinates-based ROI analysis. Two-way repeated-measures ANOVA revealed significant interaction effects (in warm colors) by acupuncture treatment on functional connectivity between the RSC and the left thalamus **(A1)**, and right SCG **(B1)**, and bilateral OFC **(C1)**. Significantly results were overlaid on the spatial maps (shown in green) of the RSC (cluster-level FWE correction). **(A2)** Average functional connectivity between the RSC and the left thalamus that showed significant interaction between group and acupuncture in the AD and NC groups. **(B2)** Average functional connectivity between the RSC and right SCG that showed significant interaction between group and acupuncture in the AD and NC groups. **(C2)** Average functional connectivity between the RSC and bilateral OFC that showed significant interaction between group and acupuncture in the AD and NC groups. Bar plots displayed mean rsFC *z* scores for the AD and NC groups. ^***^p < 0.001, ^**^p < 0.01, and ^*^p < 0.05. RSC, retrosplenial cortex; SCG, subcallosal cingulate gyrus; OFC, orbitofrontal cortex; AD, Alzheimer’s disease; NC, normal controls.

To explore the effect of acupuncture treatment by group on RSC connectivity, the interaction effect was further examined by using the aforementioned spatial map in a two-way repeated-measures ANOVA. Significant interaction effects for RSC connectivity were found and are illustrated in Figure 3. *Post hoc* pairwise comparisons demonstrated that the RSC showed significantly decreased functional connectivity with three clusters, namely, the left thalamus, the right subcallosal cingulate gyrus (SCG), and the bilateral orbitofrontal cortex (OFC), in the AD group after acupuncture treatment compared to that before acupuncture treatment (Figure 3 and Table 2). In contrast, the above FC values were significantly greater in the NC group after acupuncture treatment than in the preacupuncture group. In addition, before acupuncture treatment, the AD group exhibited significantly increased FC between the RSC and the left thalamus as well as between the RSC and right SCG relative to those in the NC group (Figures 3A1,A2,B1,B2). After acupuncture treatment, the AD group had significantly decreased FC between the RSC and the left thalamus as well as between the RSC and bilateral OFC relative to those in the NC group (Figures 3A1,A2,C1,C2).

**Table 2:** Altered RSC functional connectivity by acupuncture stimulation of Hegu (LI4) and Taichong (LIV3).

Comparison	Brain regions	Side	Cluster size	*F*/*T* value	Peak MNI coordinates		
					*X*	*Y*	*Z*
RSC_7mm							
Interaction effect	Thalamus	L	26	19.52	−6	−12	12
Interaction effect	SCG	R	62	18.35	6	18	−10
Pre_NC < Post_NC	OFC	B	126	3.70	4	62	−16
RSC_L							
Main effect	LG	L	67	13.58	−18	−54	0
Interaction effect	Thalamus	L	39	23.53	4	−4	6
Interaction effect	Thalamus	R	23	20.13	−4	−10	8
Interaction effect	PCC	L	82	12.77	−2	−52	24
Pre_NC < Post_NC	OFC	B	53	4.37	−2	50	−16
Pre_NC < Post_NC	Precuneus	R	41	3.42	4	−64	24
RSC_R							
Interaction effect	SCG	L	62	18.49	−8	16	−4
Interaction effect	OFC	B	61	17.39	−2	48	−16
Interaction effect	PCC	B	151	16.57	0	−54	18
Interaction effect	Calcarine	R	36	16.03	22	−72	14
Pre_AD < Post_AD	HPC	L	34	3.99	−20	−34	0
Pre_AD < Post_AD	PHG	R	30	3.12	28	−34	−12

Brain regions showed significant interaction effect in RSC functional connectivity between the AD and NC groups by acupuncture stimulation [Gaussian random field (GRF) corrected (voxel *p* < 0.005, cluster *p* < 0.05)]. RSC, retrosplenial cortex; SCG, subcallosal cingulate gyrus; OFC, orbitofrontal cortex; LG, lingual gyrus; PCC, posterior cingulate cortex; HPC, hippocampus; PHG, parahippocampal gyrus; L, left; R, right; B, bilateral; MNI, Montreal Neurological Institute; NC, normal controls; AD, Alzheimer’s disease.

### The effect of acupuncture on RSC connectivity in the AD and NC groups—atlas-based ROIs

**Figure 4:**
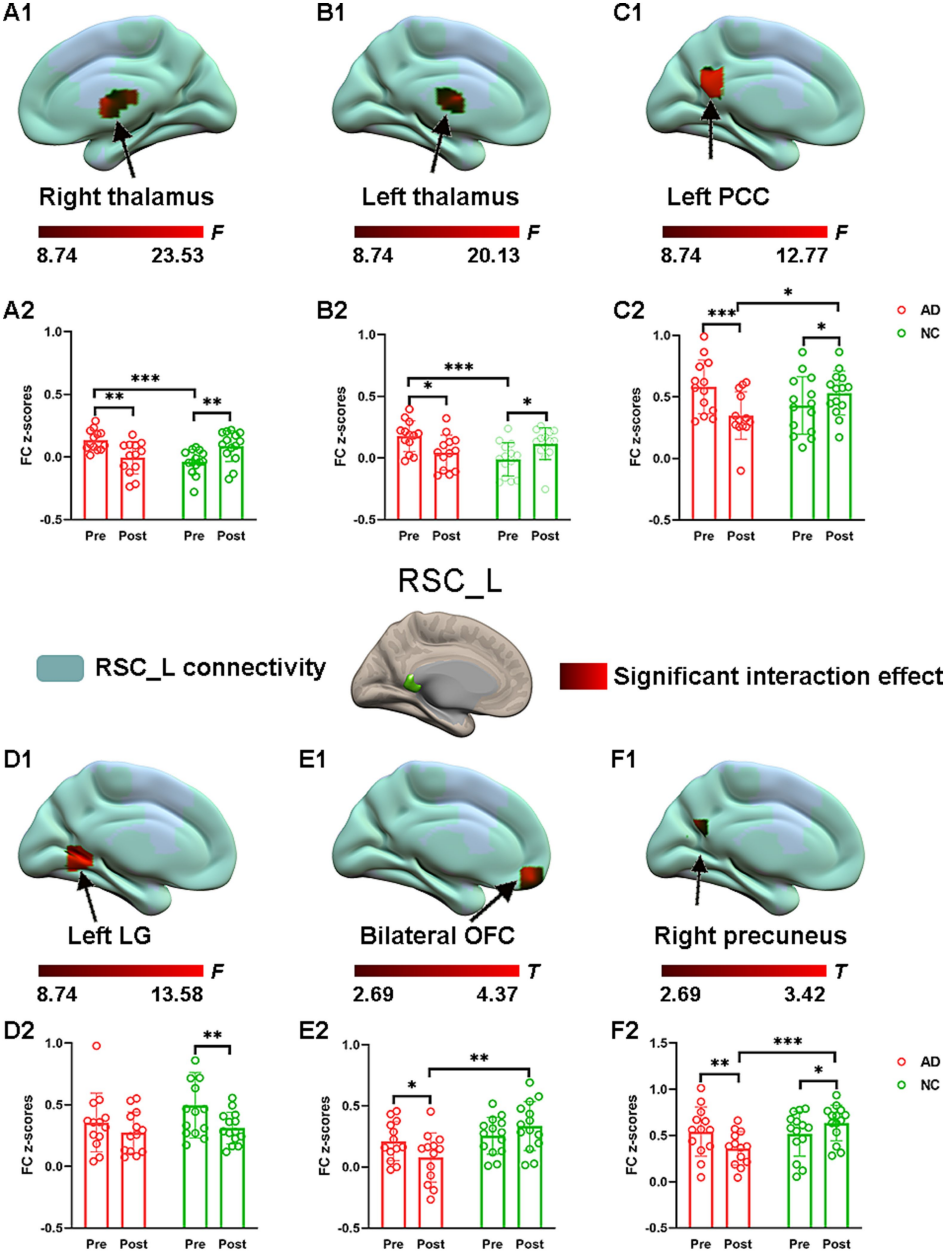
The effect of acupuncture on left RSC connectivity in the AD and NC groups as revealed by atlas-based ROI analysis. Two-way repeated-measures ANOVA revealed significant interaction effects (in warm colors) by acupuncture treatment on functional connectivity between left RSC and right thalamus **(A1)**, and left thalamus **(B1)**, and left PCC **(C1)**, and left LG **(D1)**, and bilateral OFC **(E1)**, and right precuneus **(F1)**. Significantly results were overlaid on the spatial maps (shown in green) of left RSC (cluster-level FWE correction). **(A2)** Average functional connectivity between left RSC and right thalamus that showed significant interaction between group and acupuncture in the AD and NC groups. **(B2)** Average functional connectivity between left RSC and left thalamus that showed significant interaction between group and acupuncture in the AD and NC groups. **(C2)** Average functional connectivity between left RSC and left PCC that showed significant interaction between group and acupuncture in the AD and NC groups. **(D2)** Average functional connectivity between left RSC and left LG that showed significant interaction between group and acupuncture in the AD and NC groups. **(E2)** Average functional connectivity between left RSC and bilateral OFC that showed significant interaction between group and acupuncture in the AD and NC groups. **(F2)** Average functional connectivity between left RSC and right precuneus that showed significant interaction between group and acupuncture in the AD and NC groups. Bar plots displayed mean rsFC *z* scores for the AD and NC groups. ^***^p < 0.001, ^**^p < 0.01, and ^*^p < 0.05. RSC, retrosplenial cortex; PCC, posterior cingulate cortex; OFC, orbitofrontal cortex; LG, lingual gyrus; AD, Alzheimer’s disease; NC, normal controls.

Consistent with the findings above, significant interaction effects for left RSC connectivity were also found and are illustrated in Figure 4. Specifically, *post hoc* pairwise comparisons demonstrated that compared to the preacupuncture condition, the left RSC showed significantly decreased FC with five clusters, including the bilateral thalamus, left PCC, bilateral OFC, and right precuneus, in the AD group after acupuncture treatment (Figure 4 and Table 2). In contrast, the above FCs were significantly greater in the NC group after acupuncture treatment than in the preacupuncture group. In addition, when comparing the postacupuncture condition with the preacupuncture condition, significantly decreased FC between the left RSC and left lingual gyrus was identified only in the NC group and not in the AD group (Figures 4D1,D2). Before acupuncture treatment, the FC between the left RSC and the bilateral thalamus was significantly greater in the AD group than in the NC group (Figures 4A1,A2,B1,B2). After acupuncture treatment, the AD group showed significantly decreased FC between the left RSC and the left PCC, bilateral OFC, and right precuneus relative to that in the NC group (Figures 4C1,C2,E1,E2,F1,F2).

**Figure 5:**
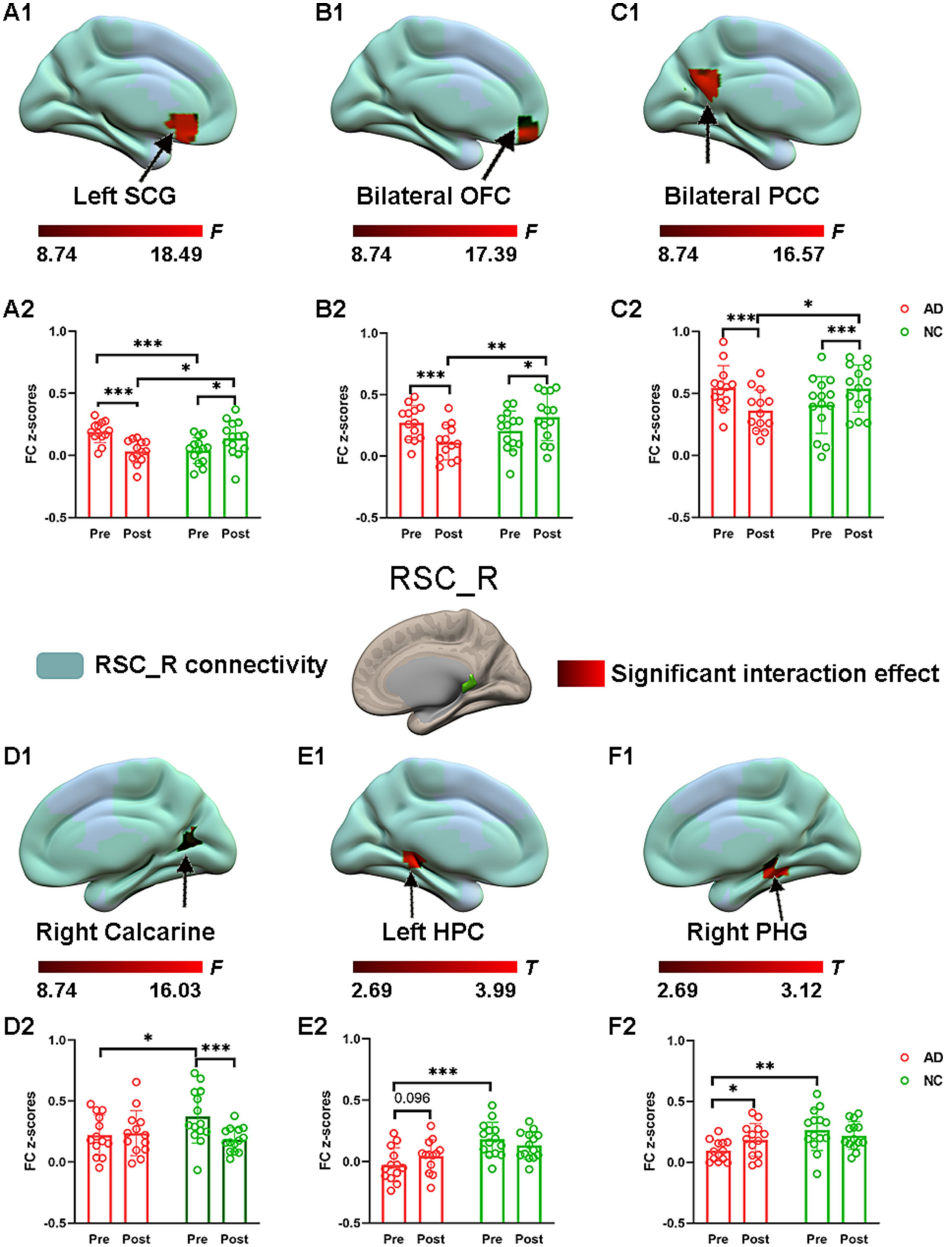
The effect of acupuncture on right RSC connectivity in the AD and NC groups as revealed by atlas-based ROI analysis. Two-way repeated-measures ANOVA revealed significant interaction effects (in warm colors) by acupuncture treatment on functional connectivity between right RSC and left SCG **(A1)**, and bilateral OFC **(B1)**, and bilateral PCC **(C1)**, and right calcarine **(D1)**, and left HPC **(E1)**, and right PHG **(F1)**. Significantly results were overlaid on the spatial maps (shown in green) of right RSC (cluster-level FWE correction). **(A2)** Average functional connectivity between right RSC and left SCG that showed significant interaction between group and acupuncture in the AD and NC groups. **(B2)** Average functional connectivity between right RSC and bilateral OFC that showed significant interaction between group and acupuncture in the AD and NC groups. **(C2)** Average functional connectivity between right RSC and bilateral PCC that showed significant interaction between group and acupuncture in the AD and NC groups. **(D2)** Average functional connectivity between right RSC and right calcarine that showed significant interaction between group and acupuncture in the AD and NC groups. **(E2)** Average functional connectivity between right RSC and left HPC that showed significant interaction between group and acupuncture in the AD and NC groups. **(F2)** Average functional connectivity between right RSC and right PHG that showed significant interaction between group and acupuncture in the AD and NC groups. Bar plots displayed mean rsFC *z* scores for the AD and NC groups. ^***^p < 0.001, ^**^p < 0.01, and ^*^p < 0.05. RSC, retrosplenial cortex; PCC, posterior cingulate cortex; OFC, orbitofrontal cortex; SCG, subcallosal cingulate gyrus; HPC, hippocampus; PHG, parahippocampal gyrus; AD, Alzheimer’s disease; NC, normal controls.

Significant interaction effects for right RSC connectivity are shown in Figure 5. *Post hoc* pairwise comparisons demonstrated that compared to the preacupuncture condition, the right RSC showed significantly decreased FC with three clusters, including the left SCG, bilateral PCC, and bilateral OFC, in the AD group after acupuncture treatment (Figure 5 and Table 2). In contrast, the above FCs were significantly greater in the NC group after acupuncture treatment than before treatment. In addition, when comparing the postacupuncture condition with the preacupuncture condition, significantly decreased FC between the right RSC and the right calcarine was identified only in the NC group and not in the AD group (Figures 5D1,D2). Significantly increased FC between the right RSC and left HPC as well as between the right RSC and the right parahippocampal gyrus was identified only in the AD group and not in the NC group (Figures 5E1,E2,F1,F2) when comparing these two conditions. Before acupuncture treatment, the FC between the right RSC and left SCG was significantly greater in the AD group than in the NC group (Figures 5A1,A2). The AD group also showed significantly decreased FC between the right RSC and right calcarine, left HPC, and right parahippocampal gyrus relative to those in the NC group (Figures 5D1,D2,E1,E2,F1,F2). After acupuncture treatment, the AD group showed significantly decreased FC between the right RSC and left SCG, bilateral PCC, and bilateral OFC relative to those in the NC group (Figures 5A1,A2,B1,B2,C1,C2).

### Correlations between FC and cognitive performance in AD patients

**Figure 6:**
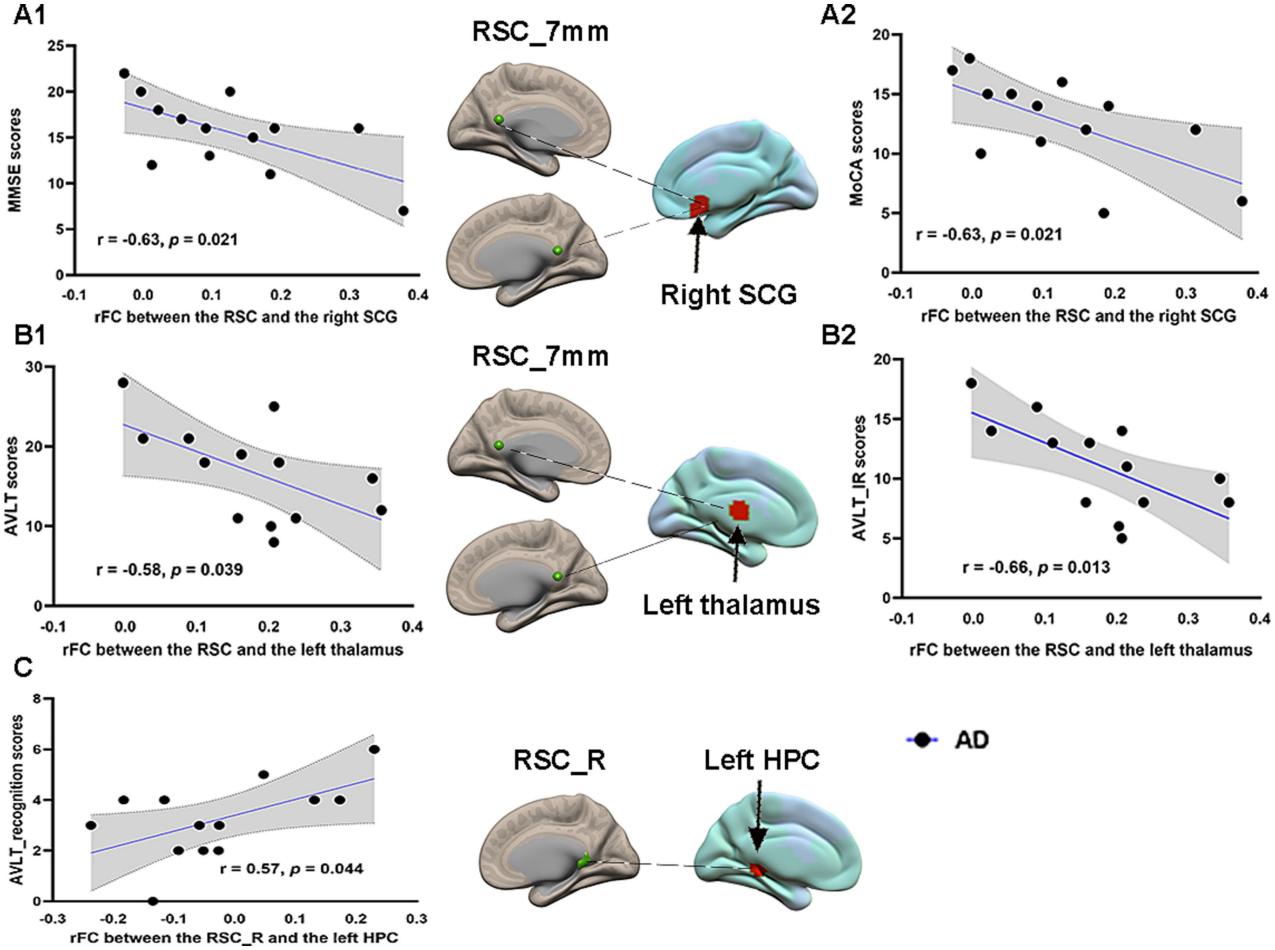
Pearson correlation analysis between the RSC FCs and neuropsychological scales in the AD group before acupuncture treatment. Brain maps of related areas are showed in the figure and colored areas represent their locations. Dashed lines are for illustrating purpose. **(A1)** The connectivity strengths between the RSC and right SCG showed a negative correlation with the MMSE scores. **(A2)** The connectivity strengths between the RSC and right SCG had a negative correlation with the MoCA scores. **(B1)** The connectivity strengths between the RSC and left thalamus showed a negative correlation with the AVLT scores. **(B2)** The connectivity strengths between the RSC and left thalamus showed a negative correlation with the AVLT_IR scores. **(C)** The connectivity strengths between right RSC and left HPC had a positive correlation with the AVLT_recognition scores. RSC, retrosplenial cortex; SCG, subcallosal cingulate gyrus; HPC, hippocampus; MMSE, Mini-Mental State Examination; MoCA, Montreal Cognitive Assessment; AVLT, Auditory Verbal Learning Test; AVLT_IR, AVLT_immediate recall; AD, Alzheimer’s disease.

Pearson correlation analysis was performed between the RSC FCs and neuropsychological scale scores in the AD group before acupuncture treatment. The significant results are summarized in Figure 6. The connectivity strengths between the RSC and right SCG were negatively correlated with the MMSE scores (*r* = −0.63, *p* = 0.021) and the MoCA scores (*r* = −0.63, *p* = 0.021) in the AD group (Figures 6A1,A2). The connectivity strengths between the RSC and left thalamus also showed a negative correlation with the AVLT scores (*r* = −0.58, *p* = 0.039) and the AVLT_IR scores (*r* = −0.66, *p* = 0.013) in the AD group (Figures 6B1,B2). Moreover, the connectivity strengths between the right RSC and left HPC were positively correlated with the AVLT_recognition scores (*r* = 0.57, *p* = 0.044) in the AD group (Figure 6C). Unfortunately, none of these associations passed correction for multiple comparisons.

## Discussion

In the current study, ROIs derived from the atlas and coordinates were used to generate a reliable connectivity map of the RSC and assess the sustained effect of acupuncture at the Si Guan points on FC with the RSC in AD patients and NCs. Some interesting results emerged from the current study. First, two sets of RSC ROIs showed robust positive connectivity with the hippocampal formation. Second, compared to the pre-acupuncture condition, multiple regions showed decreased or increased FC with the RSC after acupuncture at the Si Guan points in the AD and NC groups, which reflects the dual directional regulatory effect of acupuncture. More interestingly, for the AD patients and the NCs, acupuncture at the Si Guan points exerted opposite regulatory effects on RSC connectivity. This result was demonstrated by both coordinate-based and atlas-based ROI analyses. Third, our results demonstrated that diminished FC with the RSC was correlated with neuropsychological scale scores in the AD group before acupuncture treatment. These findings are critical for understanding the modulatory effect of acupuncture at the Si Guan points on RSC connectivity in AD patients.

Previous evidence suggests that the RSC and PCC are typically considered core functional hubs of the DMN and are involved in a broad range of cognitive functions, including episodic memory (Vann et al., 2009). However, recent fMRI studies have emphasized that the medial parietal cortex, including the PCC and RSC, is a cytoarchitectonically distinct and functionally heterogeneous region, and it is important to differentiate the RSC from the PCC due to morphological and functional variations (Vann et al., 2009; Dillen et al., 2016; Kaboodvand et al., 2018; Aggleton, 2012). More critically, the RSC has stronger structural and functional connections with the HPC than with the PCC and HPC (Kaboodvand et al., 2018; Kobayashi and Amaral, 2007). This suggests that, relative to the PCC, the RSC may be involved in hippocampus-related functions; additionally, the commutation between the RSC and the HPC may play an important role in the process of episodic memory (Vann et al., 2009; Kaboodvand et al., 2018), which has a key function in the progression of AD. Herein, our results investigating reliable FC with the RSC are consistent with these findings and demonstrated that the RSC showed robust positive connectivity with the hippocampal formation.

According to previous studies, acupuncture has a bidirectional and benign regulatory ability to restore the body’s pathological state to its normal physiological state (Cui et al., 2021; du et al., 2022). The direction of action depends on the functional state of the body. Our main findings were consistent with this theory and showed opposite regulatory effects in AD patients and NCs. Many studies have indicated that functional disconnection and compensation in brain networks coexist in individuals with AD (Wang et al., 2015; Weiler et al., 2014). Consistent with these findings, our results also revealed both decreased FC with the RSC, such as the HPC, and increased FC with the RSC, such as the thalamus and the SCG, in the AD group compared to that in the NC group before acupuncture treatment. The decreased FC with the RSC may represent functional disconnection due to the degenerative effects of the disease and increased FC with the RSC may be involved in functional compensation to preserve and compensate for losses in function. After acupuncture at the Si Guan points, a common regulatory effect on FC between the RSC and several brain regions, including the thalamus, SCG, OFC, PCC, and precuneus, was detected in both groups. However, the direction of the common regulation effect was opposite. For AD patients, the above FC with the RSC was excessively enhanced due to functional compensation, and acupuncture could deactivate the related brain regions to reduce functional connectivity with the RSC. These results are consistent with previous studies which showed increased brain activity and FC at the baseline, and reduced spontaneous neuronal activities and FC after acupuncture in brain regions located in the frontal and parietal regions, as well as in the cingulate cortex in AD patients compared with NCs, (Zheng et al., 2018; Ji et al., 2021). All these findings might be explained by the theory of dynamic functional reorganization and the dual-directional regulatory effects of acupuncture, which presented as deactivation in the increased brain activity regions in the AD patients. For normal participants, FC between the RSC and the regions above was increased by acupuncture treatment, which was consistent with previous reports showing that acupuncture can enhance resting brain networks composed of widely distributed brain areas involved in central integration, antinociception, and affective processing in healthy participants (Dhond et al., 2008; Cai et al., 2018). This modulation response may relate to acupuncture analgesia and other potential therapeutic effects. Our correlation results also support this view that enhanced FC between the RSC and SCG, as well as between the RSC and thalamus, was associated with impaired cognitive performance in AD patients before acupuncture treatment. After acupuncture, excessively enhanced FC was suppressed, which may reflect good regulation of the normal physiological state. Overall, AD patients can benefit from the common regulatory effect of acupuncture at the Si Guan points to reach homeostatic balance.

Apart from the common regulatory effect, the specific regulatory effect of acupuncture at the Si Guan points on RSC connectivity was also detected in both groups. Decreased FC between the RSC and visual cortices, such as the lingual gyrus and calcarine sulcus, was found only in the NCs after acupuncture treatment compared with baseline. Previous studies have indicated that acupuncture at the LIV3 point could specifically induce inhibition of brain areas related to vision (Ji et al., 2021; Wu et al., 2014). Our results confirmed reduced FC with visual cortical areas in healthy older adults and further revealed that acupuncture at points induces specific patterns of brain connectivity. More importantly, the current results suggested that compared with NCs, acupuncture at Si Guan points specifically elicited increased FC between the RSC and HPC as well as between the RSC and parahippocampal gyrus in AD patients. It is well known that the HPC and parahippocampal cortex are crucial for episodic memory and vulnerable to pathological changes in AD (Van Hoesen et al., 2000). Interactions between the HPC and the RSC have been demonstrated to underlie episodic memory and play critical roles in AD progression (Ziontz et al., 2021; Salami et al., 2014). Previous studies have also suggested that increased hippocampal connectivity is detected after acupuncture treatment compared with baseline in AD, which contributes to cognitive improvement (Wang et al., 2014; Zheng et al., 2018; Feng et al., 2012). Thus, it is plausible that acupuncture at Si Guan points may activate brain compensatory mechanisms in AD patients and enhance the flow of information between the RSC and medial temporal lobe, resulting in improved memory function in AD patients. Moreover, the positive correlation between the AVLT scores and FC between the RSC and HPC further supports that AD patients may recruit additional neural resources to compensate for memory loss. Collectively, all of the results demonstrated that acupuncture at the Si Guan points could exert common and specific regulatory effects on RSC connectivity to achieve better therapeutic outcomes in AD patients.

In the current study, ROIs derived from the atlas and coordinates were used to generate reliable connectivity results because the reliability of functional imaging results has been criticized in recent years (Bennett and Miller, 2010). Specifically, previous studies have proposed that the arbitrary selection of seed regions influences FC results (van den Heuvel and Hulshoff Pol, 2010; Marrelec and Fransson, 2011). Our results indicated that seed-based and atlas-based analyses yielded similar results, with atlas-based analysis yielding more comprehensive results. Using both methods, selection bias can be overcome and therefore produce an acceptable degree of reproducibility.

### Limitations

Considering these interesting findings, some limitations need to be addressed. First, the specific effect of real acupuncture could be assessed because brain activity related to sensory stimulation cannot be isolated in the current study. It would be beneficial to include a placebo control on real-needle acupuncture by using sham acupuncture, such as nonpenetrating needling, shallow needling on nonpoints, or regular needling on nonpoints. Second, although a relatively small sample size was included in this study, the current results replicated the previous findings, and reliable RSC connectivity maps were generated by using a large dataset. Future studies should increase the sample size to verify the current findings. Third, our study focused on the immediate effect of acupuncture at the Si Guan points. It is still unknown whether acupuncture at the Si Guan points could achieve a long-lasting therapeutic effect in patients with AD. In the future, a well-designed longitudinal study will be needed to trace and assess the effects of acupuncture on cognitive outcomes in AD patients at different treatment time points. Finally, based on the current consensus, early detection and intervention are key for preventing the development of AD. Therefore, future longitudinal studies are warranted to explore the potential therapeutic effects of acupuncture at the early stage of AD, such as manifestation of amnestic mild cognitive impairments.

## Conclusion

Overall, the current study provides evidence that acupuncture at the Si Guan points exerts a common and specific regulatory effect on RSC connectivity in AD patients and NCs. These findings may deepen our understanding of the bidirectional and benign regulatory mechanisms of acupuncture, and acupuncture at the Si Guan points may be a potential alternative method for the treatment of AD in the future.

## Data Availability

The raw data supporting the conclusions of this article will be made available by the authors, without undue reservation.
